# Preparation and Characterization of Sodium Alginate-Based Oxidized Multi-Walled Carbon Nanotubes Hydrogel Nanocomposite and its Adsorption Behaviour for Methylene Blue Dye

**DOI:** 10.3389/fchem.2021.576913

**Published:** 2021-03-17

**Authors:** Edwin Makhado, Mpitloane Joseph Hato

**Affiliations:** Nanotechnology Research Lab, Department of Chemistry, School of Physical and Mineral Sciences, University of Limpopo, Polokwane, South Africa

**Keywords:** sodium alginate, multi-walled carbon nanotubes, hydrogel nanocomposite, adsorption process, methylene blue 2

## Abstract

Herein, a sodium alginate/poly (acrylic acid)/oxidized-multi-walled carbon nanotubes hydrogel nanocomposite (SA/p(AAc)/*o*-MWCNTs HNC) was synthesized by *in situ* free-radical polymerization method. The synthesized SA/p(AAc)/*o*-MWCNTs HNC was used to remove methylene blue (MB) from aqueous solution. The synthesized HNC was confirmed by employing various characterization techniques. The SA/p(AAc)/*o*-MWCNTs HNC exhibited a maximum swelling capacity of 2265.4% at pH 8.0. The influence of vital parameters in the sorption process including the initial pH, adsorption dose, contact time and concentration were systematically examined on a batch mode. Subsequently, adsorption kinetics as well as isotherm models were applied to assess the nature and mechanism of the adsorption process. Adsorption kinetics were best described by pseudo-second-order model, while the Langmuir isotherm model governed the adsorption isotherm. The SA/p(AAc)/o-MWCNTs HNC exhibited a maximum adsorption capacity of 1596.0 mg/g at 25°C. This adsorbent showed excellent MB uptake and good regeneration ability.

## Introduction

Rapid industrialization has resulted in a substantial increase of dye-containing industrial effluents. The discharge of dyes into water streams give rise to negative effects on human health and the environment. Several studies reported that the exposure and indigestion of dyes can cause several health problems such as eye burn, cancer, skin irritation, nausea and dermatitis to name a few (Ngah et al., 2011; Thakur et al., 2016; Hui et al., 2018). Dyes accumulate in water due to their complex structures, which inhibit penetration of sunlight, sequentially delay photosynthesis from taking place. Consequently, it is of great importance to remove toxic dyes from wastewater. The sorption process is considered as a promising option for remediation of water mainly due to several advantages such as ease design and operation, and low cost. In this regard, various materials have been employed as adsorbents which include agricultural waste (i.e. rice husk, sawdust and bagasse) (Juang et al., 2002; Oh and Park, 2002; Malik, 2003), clay minerals such as montmorillonite, zeolites (Armağan et al., 2004; Taleb et al., 2012), chitosan and chitin (Wu et al., 2001; Crini, 2006), as well as carbon-based materials (activated carbons, multi-walled nanotubes and graphene oxide) (Ma et al., 2012; Manitha et al., 2017; Mahmoodi et al., 2018). However, the use of aforementioned materials is restricted by their partial adsorption capacity, poor reusability, production of a large amount of sludge, high operational cost and non-biodegradability (Crini and Badot, 2008; Adegoke and Bello, 2015).

The development of highly efficient, potentially sustainable and inexpensive materials for adsorption of aquatic pollutants from water is exceedingly challenging and warranted for treatment of water pollutions. Among a wide spectrum of adsorbents used for adsorption of pollutants from wastewater, hydrogels proved to be a promising adsorbents. Hydrogels represent a class of three-dimensional (3D) material consisting of cross-linked hydrophilic polymer chains, which are able to absorb and hold a large volume of liquids (Makhado et al., 2019a). Hydrogels have gained popularity among researchers due to their well-known structural features such as rapid swelling behaviour, elasticity and biocompatibility. These distinctive properties pave a way for hydrogels as potential candidates to be used in various fields including agriculture, wound dressing, drug delivery, diapers, beauty care products and wastewater treatment (Ahmed, 2015; Makhado et al., 2019b). In particular, hydrogels have spurred great interest as potential materials for adsorption of synthetic dyes and metal ions from wastewater (Pandey et al., 2020). This is due to physiochemical properties such as tunable structure, high porosity, swelling ability, fast sorption rate, and recovery capacities and reusability (El-Hag Ali et al., 2003; Shi et al., 2013). However, the applicability of hydrogels has been overshadowed due to their weak mechanical strength and partial adsorption capacity.

Given these challenges, the ongoing interest in the field of water remediation has incited the preparation of hydrogel nanocomposite with distinctive nanostructured materials. Nanomaterials including clays (Hosseinzadeh et al., 2011; Liu et al., 2018; Malatji et al., 2020), titanium dioxide (Mittal and Ray, 2016), silica (Makhado et al., 2019c), carbon-based materials (Madima et al., 2020), graphene oxide (Chen et al., 2016; Makhado et al., 2018a; Hu et al., 2020) and multi-walled carbon nanotubes (Hosseinzadeh et al., 2018) have been introduced into the hydrogel networks to synthesize hydrogel nanocomposites for wastewater remediation. Furthermore, the incorporation of these inorganic components within the polymeric hydrogel matrices can enhance the performance of a hydrogel material. In recent years, multi-walled carbon nanotubes (MWCNTs) have attracted a great deal of attention due to their unique hollow tubular structure, optical properties, excellent electrical and thermomechanical properties (Ren et al., 2011; Ma et al., 2012). However, the absence of polar functional groups on the surface of MWCNTs restricts their application in the adsorption process. Therefore, pre-treatment of MWCNTs is an ideal approach to impart polar functional groups on the surface of MWCNTs (Ma et al., 2012). Several researchers incorporated pre-treated MWCNTs in the polymer matrices and applied the prepared adsorbent for adsorption of dyes. For example, Chatterjee et al. (2010) synthesized chitosan-based MWCNTs hydrogel beads and the resultant composite was used for the removal of Congo red (CR) (Chatterjee et al., 2010). In another study, superabsorbent hydrogel nanocomposite was synthesized via graft co-polymerization of acrylamide and itaconic acid in the presence of MWCNTs by Mohammadinezhad et al. (2018). The authors reported that the addition of MWCNTs within the hydrogel networks improved mechanical properties, thermal stability and adsorption capacity toward inorganic pollutant (Mohammadinezhad et al., 2018). In another study, Makhado et al. (2018b) prepared XG/o-MWCNTs hydrogel nanocomposite and used it as an adsorbent for sequestration of methylene blue (MB) dye from aqueous medium. In their work, they reported that hydrogel nanocomposite outcompeted the hydrogel. For example, XG/o-MWCNTs hydrogel nanocomposite showed improved adsorption ability, recovery capacities and reusability, wettability and comparatively high thermal stability as compared to a bare hydrogel (Makhado et al., 2018b). It transpires from aforementioned discussions that an incorporation of the pre-treated MWCNTs in the hydrogel matrix results in the formation of hydrogel nanocomposite with improved thermal stability, adsorption capacity, as well as wettability. In our recent study, we have reported on the fabrication of sodium alginate poly(acrylic acid) (SA-poly(AA)) hydrogel and sodium alginate poly(acrylic acid)/zinc oxide (SA-poly(AA)/ZnO) hydrogel nanocomposite (HNC) via an *in situ* free-radical polymerization for the sequestration of toxic MB dye from aqueous solution (Makhado et al., 2020). In this study, we have reported that the adsorption capacity (1529.6 mg/g) for SA-poly(AA)/ZnO HNC was higher than that of the SA-poly(AA) of 1129.0 mg/g at pH 6.0 within 40 min. Interestingly, the HNC exhibited outstanding reusability with relatively better adsorption efficiencies as compared to SA-poly(AA) hydrogel. Based on the literature, we hypothesize that the incorporation of an *o*-MWCNTs in the hydrogel matrix will enhance the thermal stability, thermomechanical properties, swelling and adsorption capacity.

Herein, we report on the preparation of SA/p(AAc)/*o*-MWCNTs HNC as a potential adsorbent for adsorption of MB from aqueous solution. To elucidate the influence of *o*-MWCNTs on the formation of SA/p(AAc)/*o*-MWCNTs HNC, the structure and morphology, physicochemical characteristics (gel strength and swelling ability), thermal properties and adsorption behaviour were investigated using thermogravimetric analysis (TGA), dynamic mechanical analysis (DMA), high resolution transmission electron microscopy (HR-TEM), scanning electron microscopy (SEM), Fourier transform infrared (FTIR), Raman and X-ray diffraction (XRD). The adsorption kinetics as well as adsorption isotherm models were investigated to understand the sorption process.

## Experimental

### Materials

The biopolymer, sodium alginate (SA) having a formula weight of 176.10 g/mg and molecular formula C_6_H_8_O_6_, acrylic acid (AAc, 99 %), *N*,*N*′-methylene bis-acrylamide (MBA, 99%), ammonium persulfate (APS) (≥98%; 248614), and MWCNTs were procured from Sigma-Aldrich (South Africa). Acetone, sodium hydroxide (NaOH), nitric acid (HNO_3_, 65%), hydrochloric acid (HCl) and MB were supplied by Merck (South Africa). All chemicals used in this study were of analytical grade and used without any further purification. Deionized (DI) water was used throughout the experiment. Typically, 1.0 g of MB powder was dissolved in 1000 ml volumetric flask in DI water, stock solutions were further prepared by diluting the as-prepared stock solution from batch experiments.

### Materials Synthesis

#### Preparation of *o*-MWCNTs

Oxidation of MWCNTs was prepared according to our previously reported protocol (Makhado et al., 2018b). In brief, 0.5 g MWCNTs were weighed and added into 200 mL HNO_3_. The reaction mixture was placed in the ultrasonicator for about 10 min followed by refluxing for 12 h at 80°C under continuous stirring. The reaction mixture was allowed to cool down, then the prepared *o*-MWCNTs were washed repeatedly with DI water through filtering system. The *o*-MWCNTs were washed up until the filtrate solution reached pH 7.0, and then the obtained *o*-MWCNTs were dried in an oven.

#### Synthesis of SA/p(AAc)/*o*-MWCNTs HNC

Typically, 10 mg of SA was weighed and dissolved in 10 ml of DI water. To this mixture, appropriate amounts of APS, AAc and MBA were dissolved and added under stirring maintaining the overall volume of 30 ml. An optimum amount of *o*-MWCNTs was sonicated with 6 ml of DI water for about 5 min; this solution was then poured into 100 mL beaker reaction mixture containing SA, APS, AAc, and MBA under continuous stirring. The total volume of the reaction mixture was maintained at 30 ml. The reaction was heated at 70°C for 3 h in an oven. The product was cut to small pieces then washed with acetone followed by DI water to remove the reactant. SA/p(AAc)/*o*-MWCNTs HNC was dried in an oven at 60°C for about 24 h, subsequently milled to a fine powder.

#### Materials Characterization

Spectrum II spectrometer (Perkin-Elmer) Fourier transform infrared (FTIR) was employed to record the spectra in the wavenumber ranging between 400–4,500 cm^−1^ and resolution of 4 cm^−1^. The X-ray diffraction (XRD) patterns of the samples were collected with a PAnalytical Xpert Pro spectrometer employing Ni filtered CuKα radiation (λ = 0.1514 nm). The thermal stability was performed using a simultaneous thermal analyzer (STA) 6,000 manufactured by Perkin-Elmer (Boston, MA). The STA was connected to a PolyScience digital temperature controller under N_2_ gas purged at a flow rate of 20 ml/min. The prepared samples ranging from 1–4 mg were heated from 30–600°C at a constant heating rate of 10°C/min. The data was collected and analyzed using Pyris software®. The morphology and the elemental analysis of the prepared materials were investigated using the scanning electron microscopy (SEM) (JSM7500F, JEOL). The internal morphology of the samples was studied using high resolution-transmission electron microscopy (HR-TEM, JOEL JEM 2100, Tokyo, Japan). A dynamic mechanical analysis (DMA) was used on a single-cantilever bending mode by DMA 8000 (Perkin-Elmer) to study the mechanical stability of the synthesized hydrogels. The frequency dependence of the loss modulus and storage modulus were performed at a fixed temperature (30°C). The strain amplitude was set at 0.05%, and the ramp rate was 5°C/min in the frequency range of 0.01–100 Hz. Ultraviolet-visible spectrophotometer (UV-vis) was utilized to analyze the MB dye concentration after adsorption. The Raman spectra were recorded on a Horiba Jobin-Yvon LabRam HR 2000 confocal Raman microscope with a wavelength of 514 nm.

#### Swelling Studies

To study the hydrogel swelling ratio, 100 mg of the samples were immersed in about 80 ml of DI water at 25°C for 48 h. Thereafter, the swollen samples were removed from DI water and the excess water on the surface of the samples was wiped gently, re-weighed to determine the swelling ratio of the samples. The swelling capacity (SC) of the samples was calculated using Eq. 1.

$$SC=\frac{W_{s}-W_{d}}{W_{d}},$$ (1)

where W_d_ and W_s_ represent the weight of the initial dry sample and the weight of the swollen hydrogel sample, respectively.

#### Adsorption Studies

The sorption behavior of MB dye onto SA/p(AAc)/*o*-MWCNTs HNC was investigated on a batch mode. The adsorption studies were carried out in 30 ml of MB dye solution using 10 mg of adsorbents and agitated in a temperature-controlled shaker set at 160 rpm for a particular period. The dye-containing solutions were withdrawn and filtered with 0.45 μ PVDF syringe filters after adsorption. The influence of initial pH on the adsorption of MB was studied and the effect of pH on the adsorption capacity were ranged from 2.0 to 9.0. The influence of adsorbent dose on the adsorption capacity was varied for 10–50 mg. For adsorption kinetics studies, the contact time was altered from 10 to 60 min. For adsorption isotherm experiments, the MB dye concentrations were varied from 530 to 1,350 mg/L. The influence of contact time and equilibrium concentration was evaluated at three different temperatures 25, 35, and 45°C. After adsorption process, the aliquots were filtered and the concentration was measured using the UV-vis spectrophotometer (Cary 300) at *λ*_max_ of 662 nm for MB dye. The adsorption percentage and adsorption capacity (*q*_e_) were determined using Eqs 2,3, respectively.

$$\text{Adsorption}\left(\%\right)=\left(\frac{C_{o}-C_{e}}{C_{o}}\right)\times 100,$$ (2)

$$q_{e}\left(\frac{\text{mg}}{\text{g}}\right)=q_{t}=\left(\frac{C_{o}-C_{e}}{m}\right)V,$$ (3)

where *q*_*t*_ (mg/g) represents the amount of MB adsorbed per unit mass of the adsorbent at a certain time (*t*). *C*_*e*_ (mg/L) is the equilibrium concentration f MB, *m* (g) is the mass of the adsorbent, and *V* (L) is the volume of the MB dye solution.

#### Sorption Kinetics

To evaluate the underlying mechanism and to govern the rate constant of the sorption process the pseudo-first-order (Eq. 4) (Lagergren, 1898) and the pseudo-second-order (Eq. 5) (Ho, and McKay, 1998) were applied. Their linear forms of the former and the later equations are provided in Eqs 4,5, respectively;

$$\log\left(q_{e}-q_{t}\right)=\log\left(q_{e}\right)-\frac{k_{1}}{2.030}t,$$ (4)

$$\frac{t}{q_{t}}=\frac{1}{k^{\prime }q_{e}^{2}}+\left(\frac{1}{q_{e}}\right)t,$$ (5)

where, *q*_e_ (mg/g) is the amount of MB adsorbed at equilibrium, *q*_t_ (mg/g) is the amount of MB adsorbed at any time *t* and equilibrium, *k*′_1_(min^−1^), k′_2_ (g mg^−1^ min^−1^) are pseudo-first-order and pseudo-second-order rate constants, respectively. For pseudo-first-order model, the straight-line plot of $\log\left(q_{e}-q_{t}\right)$ against t gives log(*q*_e_) and intercept equal to k′_1_/2.303. Thus, the amount of solute sorbed per gram of sorbent at equilibrium (*q*_e_) and k′_1_ can be evaluated from the slope and the intercept.

### Adsorption Isotherm Studies

#### The Langmuir Isotherm Model

The Langmuir isotherm model is based on the monolayer adsorption of adsorbate and assumed that the adsorption takes place at the specific homogeneous adsorbent surface (Langmuir, 1918), and this is presented in Eq. 6;

$$q_{e}=\frac{q_{m}bC_{e}}{1+bC_{e}}.$$ (6)

The Langmuir equation can be rearranged to a linear form for the convenience of plotting and determination of its constant (*K*_L_) as provided Eq. 7;

$$\frac{C_{e}}{q_{e}}=\frac{1}{q_{m}b}+\frac{1}{q_{m}}\;C_{e},$$ (7)

where *q*_m_ is the monolayer adsorption capacity per unit of adsorbent (mg/g), *q*_e_ is the adsorption capacity at equilibrium (mg/g), and *b* relates the heat of adsorption (L/mg). *C*_e_ is the concentration of MB solution at equilibrium (mg/l). The values of *q*_m_ and *K*_L_ can be determined from the linear plot of *C*_e_/*q*_e_ against *C*_e_. The essential features of the Langmuir isotherm can be employed to predict the affinity between the sorbate and the sorbent using the dimensionless equilibrium constant (*R*_L_) (Makhado et al., 2017b). which can be expressed in Eq. 8:

$$R_{L}=\frac{1}{1+K_{L}C_{o}},$$ (8)

where *R*_L_ value reveals the shape and feasibility of the sorption isotherm. This parameter indicates favourable sorption if 0 < R_L_ < 1, unfavorable (*R*_L_ > 1), linear (*R*_L_ = 1), and irreversible if *R*_L_ = 0.

#### Freundlich Isotherm Model

The Freundlich isotherm model is valid for the multilayer adsorption of adsorbate at the heterogeneous adsorbent surface (Freundlich, 1906). Furthermore, the adsorption capacity depends upon the concentration of adsorbate. The Freundlich isotherm is generally expressed by the following empirical Eq. 9:

$$q_{e}=K_{F\;}C_{e}^{\frac{1}{n}}.$$ (9)

The linear expression takes the following form given in Eq. 10:

$$\log\;q_{e}=\log\;K_{f\;}+\left(\frac{1}{n}\right)\;\log\;C_{e},$$ (10)

where *K*_F_ is the Freundlich equilibrium constant (mg/g(L/mg)^1/n^), *C*_e_ is the equilibrium concentration (mg/l), and the parameter 1/n is the adsorption heterogeneity constant (varies with the nature of the adsorbent/adsorbate system) and *q*_e_ is the amount of adsorbate adsorbed per unit adsorbent at equilibrium (mg/g). Both K_F_ and 1/n are evaluated from the intercept and the slope respectively, of the linear plot of log $q_{\text{e}}$ against logC_e_.

#### Temkin Isotherm Model

The Temkin isotherm model takes into consideration the adsorbate-adsorbent interaction (Temkin, 1941). This model also suggests that the heat of adsorption decreases linearly with the increase in adsorption quantity. The model is provided by Eqs 11–13 as follows:

$$q_{\text{e}}=\frac{RT}{{\text{b}}_{T}}\ln{\text{A}}_{T}+\frac{RT}{{\text{b}}_{T}}\text{ln}C_{\text{e}},$$ (11)

$$\text{b}=\frac{\text{RT}}{{\text{b}}_{T}},$$ (12)

$$q_{\text{e}}={\text{β ln A}}_{\text{T}}+{\text{βlnC}}_{\text{e}},$$ (13)

where *b*_T_ is Temkin isotherm constant, A_T_ is the Temkin isotherm equilibrium binding constant (L/g), *β* is constant related to the heat of adsorption (J/mol), *R* is the universal gas constant (8.314 J/mol/K) and *T* is the temperature at (273.15 K).

#### Determination of Point of Zero Charge

The point of zero charge (pH_pzc_) is simply the pH at which the adsorbent surface has a net charge of zero. pH_pzc_ is obtained from the difference between the initial and the final pH (∆pH). To determine the pH_pzc_, measured amounts of the hydrogel and the hydrogel nanocomposite were immersed in aqueous solutions of pH ranging between 2.0–10.0 and agitated in a shaker (170 rpm, ambient temperature) for 48 h. The solution pH was recorded and the pH_pzc_ was calculated using Eq. 14:

$$\Delta pH=pH_{final}-pH_{initial}.$$ (14)

#### Statistical Analysis

The root-mean-squared error (RMSE) was measured to assess how well the adsorption kinetics and isotherm model performed. The RMSE is calculated using the following Equation:

$$\text{RMSE}=\sqrt{\overset{\text{N}}{\underset{\text{i}=1}{\sum }}\frac{{\left({\text{q}}_{\text{exp}}-{\text{q}}_{\text{mod}}\right)}^{2}}{n}}$$ (15)

where q_exp_ is the equilibrium capacity, q_mod_ denotes the model predictions of equilibrium capacity and *n* is the number of experimental data points (Sun et al., 2010; Despotovic et al., 2016; Matome et al., 2020).

#### Desorption and Regeneration Studies

Reusability of samples was studied through repeated adsorption-desorption experiments. The MB-loaded adsorbents were recovered and recycled by rinsing with 0.1 M HCl solution and agitated at 170 rpm for 3 h, then neutralized with 0.1 M NaOH solution followed by DI water. The absorbent were dried at 50°C, pulverized, and reused in the next cycle of the adsorption. The dye adsorption/desorption cycles were repeated six times and calculated as given in Eq. 16.

$$\text{Desorption}\;\text{percentage}\left(\text{\%}\right)=\frac{\text{Concentration}\;\text{desorbed }\left(\text{mg}/\text{l}\right)}{\text{Concentration}\;\text{adsorbed }\left(\text{mg}/\text{l}\right)}\times 100.$$ (16)

## Results and Discussions

### FTIR, Structural and TGA Studies

**FIGURE 1:**
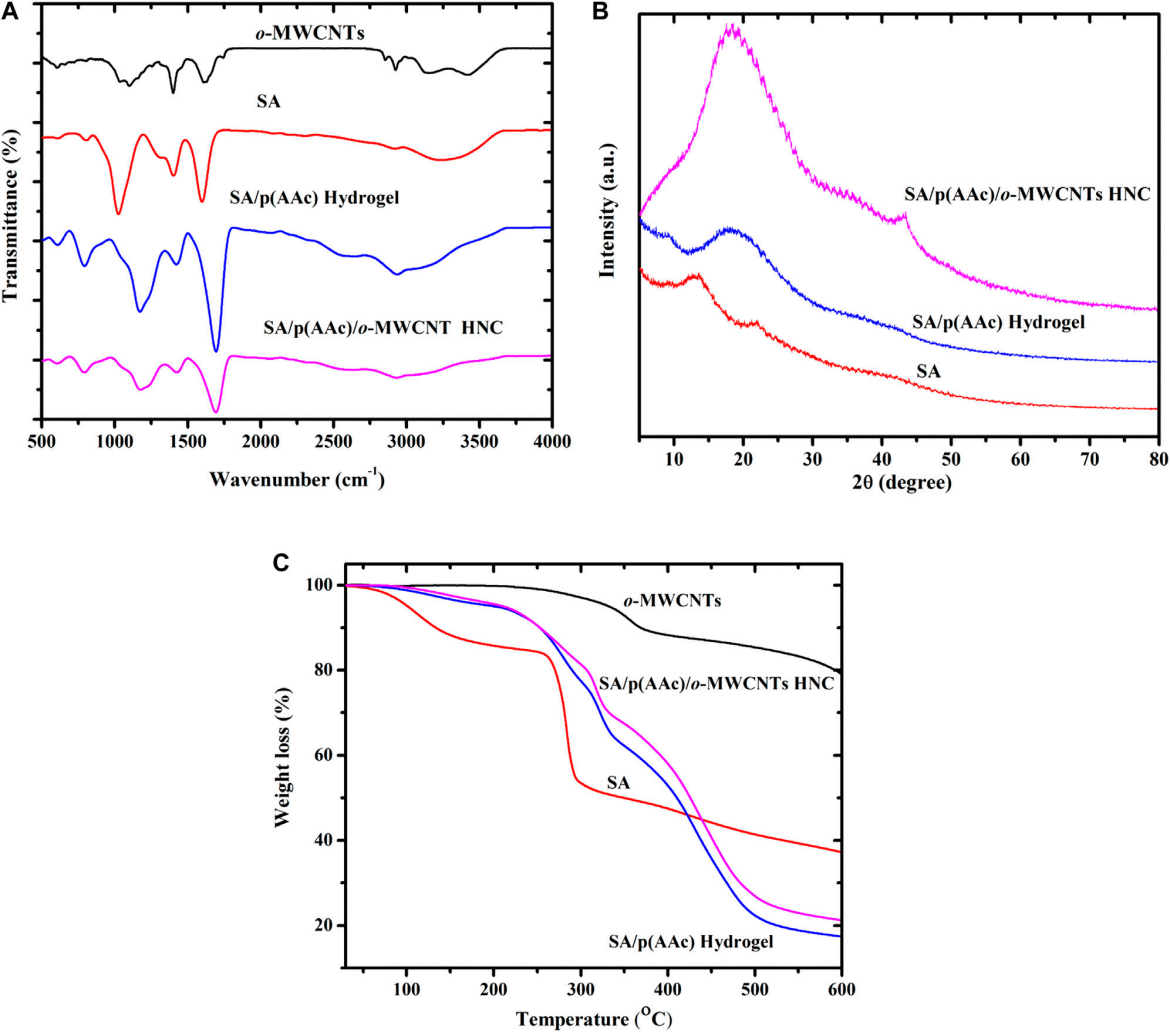
**(A)** FTIR, **(B)** XRD spectra and **(C)** TGA thermograms of *o*-MWCNTs, SA, SA/p(AAc) hydrogel and SA/p(AAc)/*o*-MWCNTs HNC.

The FTIR spectra of SA, *o*-MWCNTs, SA/p(AAc) hydrogel and SA/p(AAc)/*o*-MWCNTs HNC are shown in Figure 1A. It is noticeable that *o*-MWCNTs displayed a broad characteristic peak at 3,666 cm^−1^ to 3,015 cm^−1^, corresponding to the–OH groups. The sharp peaks at 2,922 cm^−1^ and 2,841 cm^−1^, attributed to the asymmetric stretching and symmetric vibrations of CH_2_ group (Dyke and Tour, 2004). The characteristic bands corresponding to C-O and–COO groups at 1,035 cm^−1^ and 1608 cm^−1^, respectively. The spectrum of SA revealed a wide band between 3,100 and 3,620 cm^−1^ related to the stretching vibration of the hydroxyl groups present in the SA polymer chain. The peak at 2,922 cm^−1^ was assigned to the –CH, which stretching vibrations of the CH_2_ (Makhado et al., 2017a; Makhado et al., 2017b). Two absorption peaks at about 1,608 and 1,407 cm^−1^ were ascribed to the COO^−^ groups and the C-C bending vibration, respectively. The band at 1,034 cm^−1^ emanated from the C-O-C stretching frequencies (Hu et al., 2018; Wattie et al., 2018). The characteristic peak at 802 cm^−1^ relates to the Na-O of the SA (Thakur et al., 2016; Olad et al., 2018). After graft co-polymerization of AAc onto SA, an increase in the peak intensity of SA was observed at 802 cm^−1^ which was attributed to the β-glycosidic linkage between the guluronic units and a very broad absorption peak appeared at 3,660–2,500 cm^−1^ due to carboxylic acid OH stretch. Furthermore, the shift in the position of the characteristic peaks of SA shifted from 1,680 to 1,700 cm^−1^ which is possibly due to the strong interaction with AAc and the stretching of the C-O-C bond shifted from 1,023 to 1,121 cm^−1^ which confirms the SA/p(AAc) hydrogel formation (Thakur et al., 2016). In the spectrum of SA/p(AAc)/*o*-MWCNTs HNC, the characteristic peaks emanating from SA/p(AAc) hydrogel were all observed with a reduction in the intensity after the formation of hydrogel nanocomposite, these results suggest that the internal hydrogen bonding occurred between the *o*-MWCNTs and SA/p(AAc) hydrogel.

The XRD patterns of pure MWCNTs and *o*-MWCNTs are illustrated in Supplementary Figure S1B. Both samples showed three diffraction peaks at 2*θ* = 25.8° relating to the (002) plane of the graphitic carbon (JCPDS-ICDD No. 751621), 2*θ* = 43.1° corresponding to the (001) and 2*θ* = 53.3° belonging to the (004) reflections of the carbon atoms. The diffraction patterns of samples exhibited the same trend without any shift in the 2*θ* peak positions, which indicate that the microstructure of MWCNTs was not destroyed during oxidizing. Moreover, the intensity of diffraction peaks of the *o*-MWCNTs spectrum was slightly higher than that of pure MWCNTs, confirming that pure MWCNTs were oxidized (Supplementary Figure S1B). These observations were in good agreement with the results obtained from Raman studies (Supplementary Figure S1A). XRD spectra of pure SA, SA/p(AAc) hydrogel and SA/p(AAc)/*o*-MWCNTs HNC are displayed in Figure 1B. In the case of pure SA, 2*θ* diffraction peaks at 13.8°, 21.5°, and 39.1° can be attributable to the reflection of (110) plane from polyguluronate unit, (200) plane from polymannuronate and the latter from amorphous halo, respectively. After graft copolymerization of AAc onto SA backbone, the peaks emanating from SA emerged and formed a broad diffraction peak at 2*θ* = 18.9°, without any clear diffraction peaks confirming the amorphous nature of SA/p(AAc) hydrogel. The disappearance of two diffraction peaks suggested successful grafting of AAc onto SA backbone. Several studies showed that grafting of AAc onto biopolymer results in the interference of the backbone diffraction peaks (Thakur et al., 2016; Verma et al., 2020). Subsequent SA/p(AAc)/*o*-MWCNTs HNC formation, the appearance of a wide broad peak with higher intensity was observed at 2*θ* = 18.9° and a diffraction peak at 2*θ* = 43.2° corresponding to (100) reflection, which may be due to a short-range order in *o*-MWCNTs layers. The XRD results are in good agreement with the FTIR results, confirming the formation of SA/p(AAc) hydrogel as well as SA/p(AAc)/*o*-MWCNTs HNC.

The TGA of *o*-MWCNTs, SA, SA/p(AAc) hydrogel and SA/p(AAc)/*o*-MWCNTs HNC is shown in Figure 1C. The thermal characteristics of the *o*-MWCNTs showed high stability over the studied range 25–600°C. It was observed that SA exhibits two degradation steps. In the first step, weight loss of about 10% occurred between 50 and 180°C which may be attributed to dehydration of SA and volatilization of water. The major weight loss of around 40% occurred over the range 250–300°C, which was mainly due to the SA backbone decomposition. The thermal characteristics of SA/p(AAc) hydrogel and SA/p(AAc)/*o*-MWCNTs HNC demonstrated similar degradation profiles with curves disintegrated in three steps. The first degradation of 5% occurred in the temperature range of 90–210°C due to the loss of water residual. In the temperature region of 250–350°C, there was a second decomposition stage of about 20% due to degradation of SA and residue carbide form (Soares et al., 2004). In the third degradation profile, about 50% weight reduction occurred in the temperature range of 330–500°C due to the breaking of its carboxyl from acrylic acid groups and degradation of the residual copolymer. Overall, the correlation analysis demonstrated that SA/p(AAc)/*o*-MWCNTs HNC had improved thermal stability as compared to SA/p(AAc) hydrogel. The presence of *o*-MWCNTs in the hydrogel matrix enhanced the thermal property of the hydrogel.

### Morphological Characteristics

**FIGURE 2:**
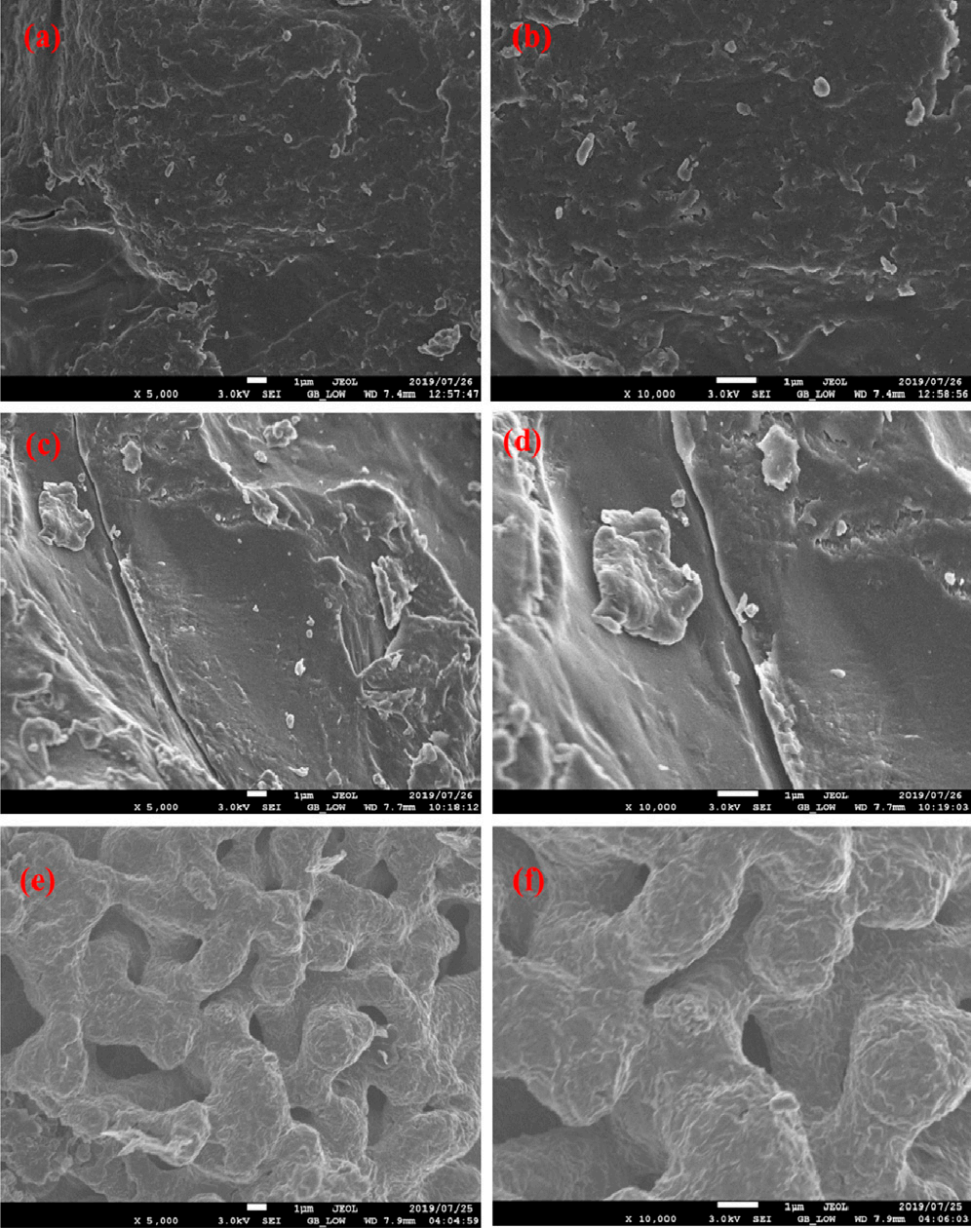
SEM images of **(A, B)** SA **(C, D)** SA/p(AAc) hydrogel **(E, F)** SA/p(AAc)/o-MWCNTs HNC.

Structural morphologies of SA, SA/p(AAc) hydrogel and SA/p(AAc)/*o*-MWCNTs HNC was subjected to SEM analysis. Figures 2A,B depicts low and high magnification of SA. Fragment-like loose surface was observed for the pure SA, which is indicative of semi-crystalline and amorphous structure. After the formation of the three-dimensional polymer network, the surface morphology presented a relatively continuous coarse surface which is a consequence of the presence of AA (Figures 2C,D). Figures 2E,F illustrated the low and high magnification of SA/p(AAc)/*o*-MWCNTs HNC. It was observed that subsequent hydrogel nanocomposite formation, the surface morphology of became utterly different from the continuous coarse surface to rough, irregular and porous structure. The rough, irregular and porous structure results from interactions between *o*-MWCNTs and polymeric hydrogel matrices.

**FIGURE 3:**
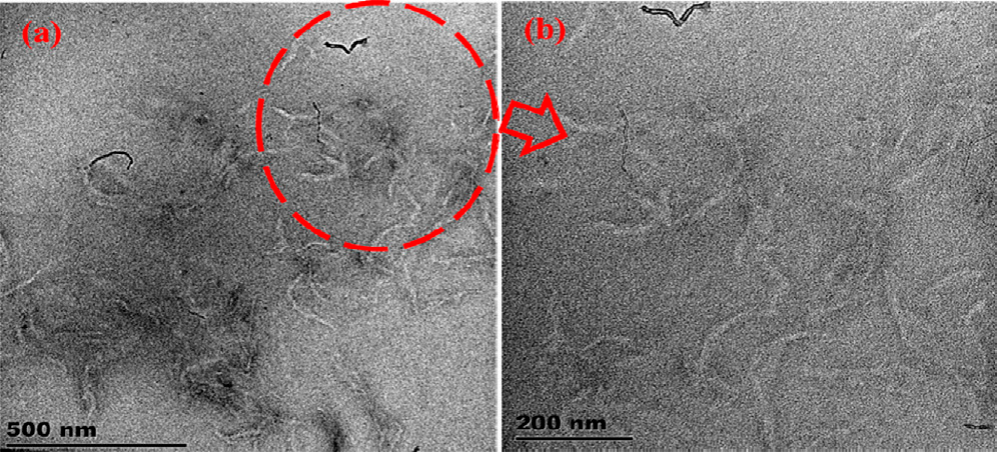
TEM images of **(A)** and **(B)** SA/p(AAc)/*o*-MWCNTs HNC.

To get information on the homogeneity of *o*-MWCNTs on the SA/p(AAc) hydrogel matrix, TEM analysis was performed and the results are shown in Figures 3A,B. From the micrographs, it is seen that *o*-MWCNTs were mostly uniformly dispersed on the SA/p(AAc) hydrogel matrix to form SA/p(AAc)/*o*-MWCNTs HNC. The *o*-MWCNTs were compatible with the polymeric matrices due to availability of hydrophilic functional groups, which promote strong H bonding interactions.

### Mechanical Properties

**FIGURE 4:**
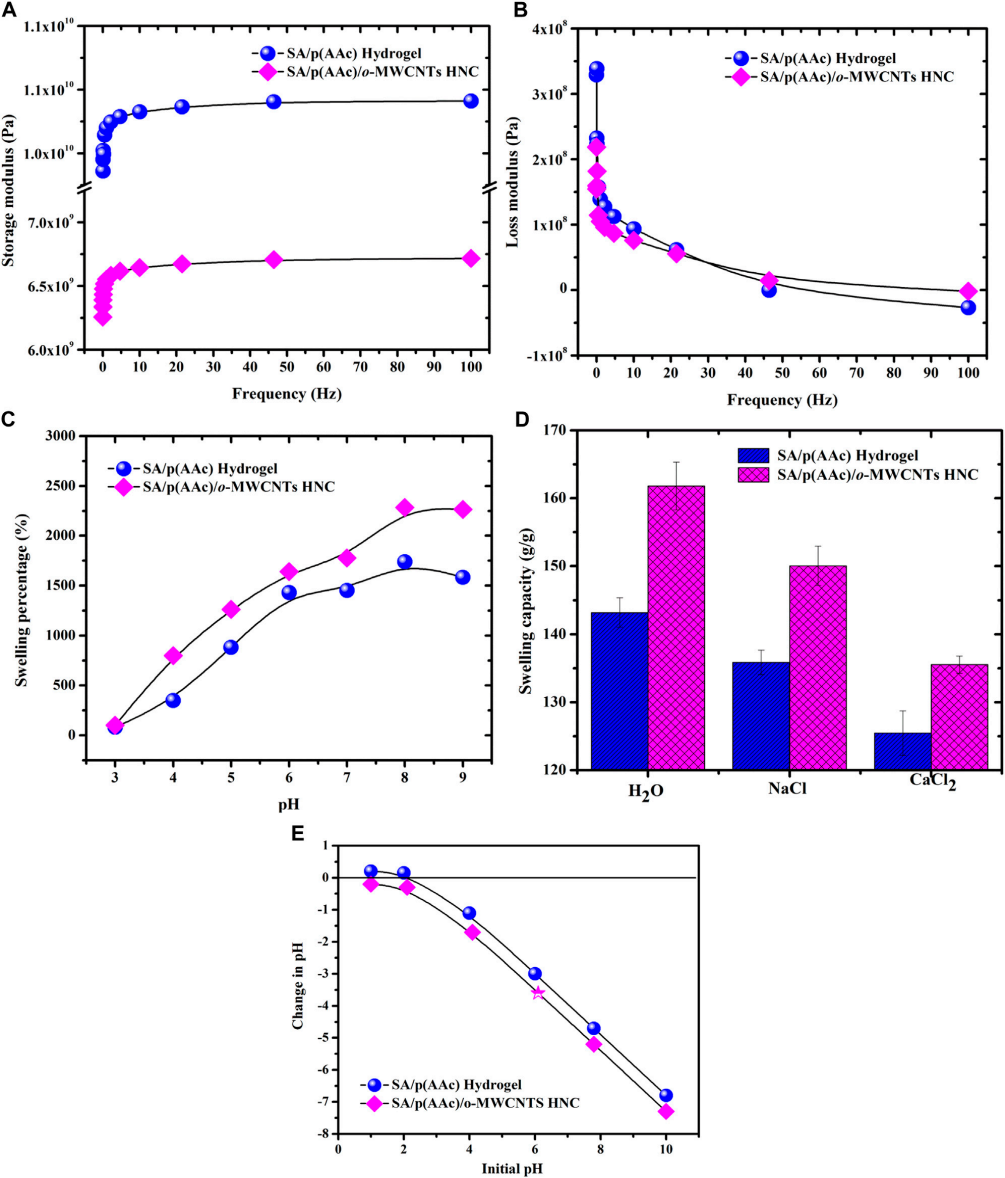
DMA of SA/p(AAc) hydrogel and SA/p(AAc)/*o*-MWCNTs HNC; **(A)** storage modulus, **(B)** loss modulus, **(C)** effect of pH on the swelling percentage, **(D)** effect of salts on the swelling capacity and **(E)** pH_PZC_.

The viscoelastic property of polymeric hydrogel is an important factor which affects their applications. The storage modulus, G′ (elastic response) and loss modulus, G″ (viscous behavior) of the SA/p(AAc) hydrogel and SA/p(AAc)/*o*-MWCNTs HNC were measured as a function of frequency using DMA. Figure 4A shows the G′ of SA/p(AAc) hydrogel and its nanocomposite in the 0.01–100 Hz frequency range and similar trends were noticed. It can be seen that both SA/p(AAc) hydrogel and SA/p(AAc)/*o*-MWCNTs HNC exhibited elastic character at low frequencies. The SA/p(AAc)/*o*-MWCNTs HNC revealed lower elastic response than SA/p(AAc) hydrogel. This result suggests that the incorporation of *o*-MWCNTs in the polymeric hydrogel matrices weakened the degree of cross-linking density and caused the decrease in G′. Figure 4B displays the viscous behavior of SA/p(AAc) hydrogel and SA/p(AAc)/*o*-MWCNTs HNC as a function of frequency. The G″ of SA/p(AAc) hydrogel was slightly higher than that of SA/p(AAc)/*o*-MWCNTs HNC in the frequency range of 0.01–30 Hz, cross over was noticed at 30 Hz. As the frequency of the system was increased above the characteristic frequency of the segments, the dissipative component increased. It can be observed from Figures 4A,B that the storage modulus was greater than the loss modulus throughout the studied frequency range, which was indicative of elastic character.

### Swelling Studies

The water uptake of SA/p(AAc) hydrogel and SA/p(AAc)/*o*-MWCNTs HNC was investigated in the pH range 3.0–9.0 (Figure 4C). The swelling studies results demonstrated that both adsorbents had pH-dependent behavior. Moreover, both adsorbents showed similar trends for the water uptake throughout the studied pH range. In acidic conditions, both adsorbents displayed the lowest swelling degree. It was observed that the increase in the solution pH increased the swelling capacity of SA/p(AAc) hydrogel and SA/p(AAc)/*o*-MWCNTs HNC. The maximum swelling percentage of 1,669.7 and 2,268.4% was observed at pH 8.0 for SA/p(AAc) hydrogel and SA/p(AAc)/*o*-MWCNTs HNC, respectively. In the alkaline pH, the network structure of both adsorbents exhibited extensive swelling in the aqueous medium due to the presence of hydrophilic groups, i.e., -COOH, which renders a negative charge density that participates in the electrostatic interactions with the water molecules. The *o*-MWCNTs incorporated hydrogel nanocomposite exhibited greater swelling capacities than the SA/p(AAc) hydrogel. The increment in the swelling degree of SA/p(AAc)/*o*-MWCNTs HNC can be ascribed to the addition of hydrophilic *o*-MWCNTs in the hydrophilic polymeric matrices (Makhado et al., 2018b). The swelling degree of SA/p(AAc)/*o*-MWCNTs HNC was comparatively high, which is owing to the low gel strength. It is well proven that the improvement in the gel strength is inversely proportional to the swelling degree (Thakur et al., 2016). Therefore, the swelling studies results support the mechanical studies results.

### Effect of Ionic Strength on the Swelling Capacity

The influence of salts (0.1 M of NaCl and CaCl_2_) on the swelling capacity of SA/p(AAc) hydrogel and SA/p(AAc)/*o*-MWCNTs HNC was investigated and the results are shown in Figure 4D. It was observed that both adsorbents exhibited high swelling capacity in the absence of salts. The maximum swelling capacity of 136.0 and 150.1 g/g for SA/p(AAc) hydrogel and SA/p(AAc)/*o*-MWCNTs HNC was recorded for the solution of NaCl, respectively. In the solution of CaCl_2_, the highest water uptake was found to be 124.8 and 149.7 g/g for SA/p(AAc) hydrogel and SA/p(AAc)/*o*-MWCNTs HNC. These observations demonstrate that salts reduced the water molecule adsorption efficiency of both adsorbents. These reductions may be attributed to the competition between water molecules and the surface of the adsorbent. The pH value at the point of zero charges (pHpzc) on the SA/p(AAc) hydrogel surface was found to be 2.2 and the surface become negatively charged with increasing pH values. On the other hand, SA/p(AAc)/*o*-MWCNTs HNC demonstrated negatively charged surface in the studied pH range and the negative charge on the surface of SA/p(AAc)/*o*-MWCNTs HNC increased with increasing pH values (Figure 4E). These results may be explained by dissociation of the hydroxyl groups that imparts the negative charge to the MWCNTs surface. The negatively charged surface promote the adsorption of positively charged adsorbate such as MB. A similar behaviour was reported in the literature (Makhado et al., 2018b).

### Adsorption Studies

Various factors influencing the adsorption process such as solution pH, adsorption dose, contact time and initial dye concentration in the solution must be optimized for application in real-world situations.

### Effect of Initial Solution pH on the Adsorption of MB Dye

**FIGURE 5:**
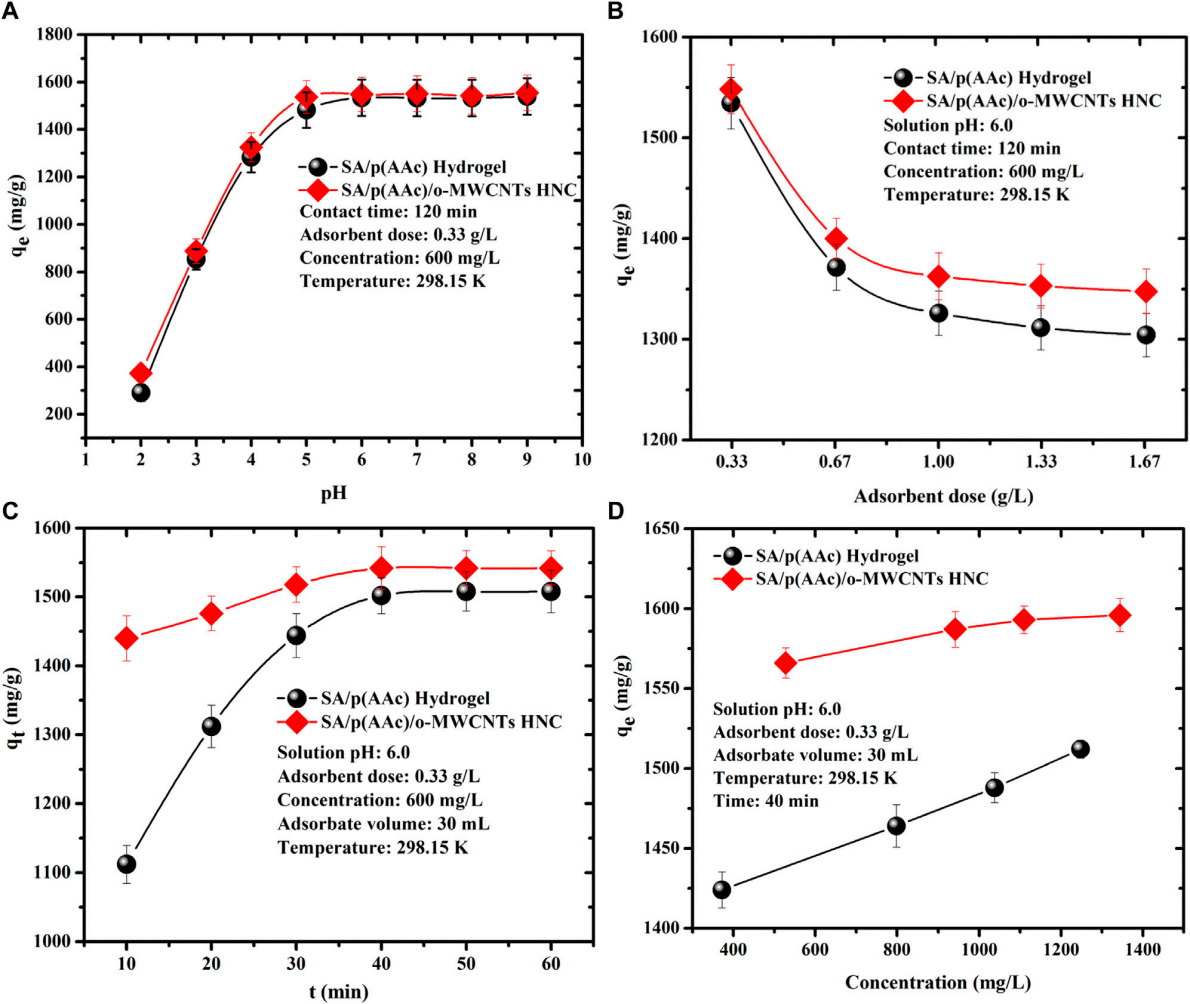
Effect of **(A)** initial pH, **(B)** adsorbent dose, **(C)** contact time, and **(D)** equilibrium concentration on the adsorption capacity of MB dye.

The influence of solution pH plays a significant role in the adsorption process. The solution pH governs the interaction between the ions present in the reaction system and the adsorbent surface. Figure 5A showed that the adsorption capacity increased with the increasing solution pH and subsequently the equilibrium was reached at pH 6.0. FTIR results confirmed that SA/p(AAc)/*o*-MWCNTs HNC has carboxylic groups which could be attributed to the high MB affinity at pH above 5.0. In an acidic medium, the carboxylic groups on the SA/p(AAc)/*o*-MWCNTs HNC surface were protonated limiting MB uptake due to columbic repulsion. With an increase in the solution pH, electrostatic interaction between negatively charged SA/p(AAc)/*o*-MWCNTs HNC surface and cationic MB dye molecules enhanced the adsorption capacity. In a basic medium, the high adsorption capacity was obtained due to ionization of carboxylic groups at higher pH, favoring intermolecular interaction between adsorbate and adsorbent. Therefore, the optimum solution pH used for the adsorption of MB in this study was 6.0. Similar observations were noticed with SA/p(AAc) hydrogel.

### Effect of Adsorbent Dose on the Adsorption of MB Dye

It is conspicuous in Figure 5B that the adsorption capacity was decreased with the increasing the amount of SA/p(AAc) hydrogel and SA/p(AAc)/*o*-MWCNTs HNC in the adsorption process. The adsorption capacity of SA/p(AAc)/*o*-MWCNTs HNC decreased from 1,540 to 1,350 mg/g as the adsorbent dose was increased from 0.33 to 1.67 g/l at a fixed solution pH, contact time and equilibrium concentration. The high adsorption capacity was achieved at low adsorbent dose due to complete occupation of the active sites. At high adsorbent loading, the adsorption capacity decreased due to less occupation of adsorption active sites and agglomeration of the particles in the reaction mixture. SA/p(AAc)/*o*-MWCNTs HNC showed a high adsorption capacity as compared to the neat SA/p(AAc) hydrogel throughout the studied range.

### Effect of Contact Time and Adsorption Kinetics

**FIGURE 6:**
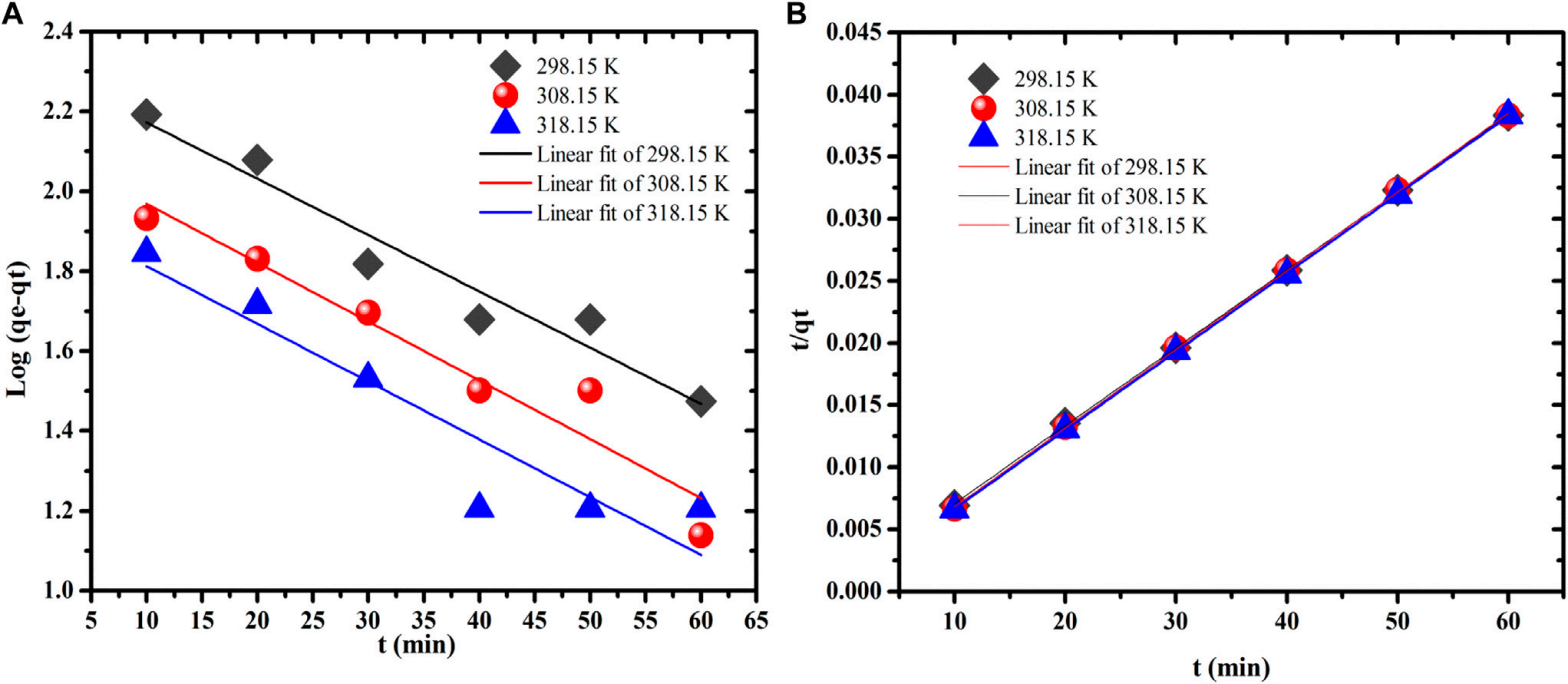
Adsorption kinetic plots for MB dye onto SA/p(AAc)/*o*-MWCNTs HNC **(A)** pseudo-first-order and **(B)** pseudo-second-order model.

The influence of contact time on the adsorption capacity of MB by SA/p(AAc) hydrogel and SA/p(AAc)/*o*-MWCNTs HNC was evaluated in the range of 10–50 min at solution pH of 6.0, 0.33 g/l adsorbent dose and 600 mg/l at three different temperatures. As depicted in Figure 5C, the adsorption capacity of MB was rapid and then, attained equilibrium within 40 min. The adsorption kinetic models were applied to investigate the adsorption mechanism of adsorbent and adsorbate. To better understand the adsorption process, the experimental data in Supplementary Figure S2C were evaluated using two common kinetic models namely pseudo-first-order and pseudo-second-order. As shown in Figures 6A,B and Table 1, the pseudo-second-order displays the correlation coefficient (R^2^) of 0.999, which is higher than that of the pseudo-first-order model. Moreover, the calculated RMSE values favored the pseudo-second-order model as a probable kinetic model. These results and kinetic theory, suggest that MB adsorption onto SA/p(AAc)/*o*-MWCNTs HNC was governed by chemisorption and physicochemical interactions (Makhado et al., 2019b).

**TABLE 1:** Adsorption kinetic model parameters for MB removal by SA/p(AAc)/*o*-MWCNTs HNC at three different temperatures. Influence of initial MB concentration and adsorption isotherm studies.

Kinetic models	Parameters	298.15 (K)	308.15 (K)	318.15 (K)
Pseudo-first-order	K^′^_1_ (min^−1^)	0.0141	0.0147	0.0145
	R^2^	0.9389	0.9192	0.8555
	RMSE	0.1067	0.0145	0.0107
Pseudo-second-order	q_e (cal.)_ (mg/g)	1595.8	1579.8	1581.8
	K^′^_2_ (g mg^−1^ min^−1^)	0.0008	0.0005	0.0004
	R^2^	0.9998	0.9998	0.9999
	RMSE	0.00006	0.00005	0.00002

The effect of the of initial concentration MB dye on the adsorption capacity of the SA/p(AAc)/*o*-MWCNTs HNC was evaluated in the range 530–1,350 mg/L of under optimum conditions (pH 6.0, 50 min contact time and a dose of 0.33 g/L) at three different temperatures, and the results are presented in Supplementary Figure S2D. It was observed that the adsorption capacity of SA/p(AAc)/*o*-MWCNTs HNC increased with increasing temperature, which implied that the adsorption process was endothermic in nature. The increase in adsorption capacity with increasing initial concentration demonstrated that the mass gradient functions as the driving force for the adsorption process. Furthermore, an increase in the concentration of MB dye solution at fixed SA/p(AAc)/*o*-MWCNTs HNC amount raised the mass transfer process.

**FIGURE 7:**
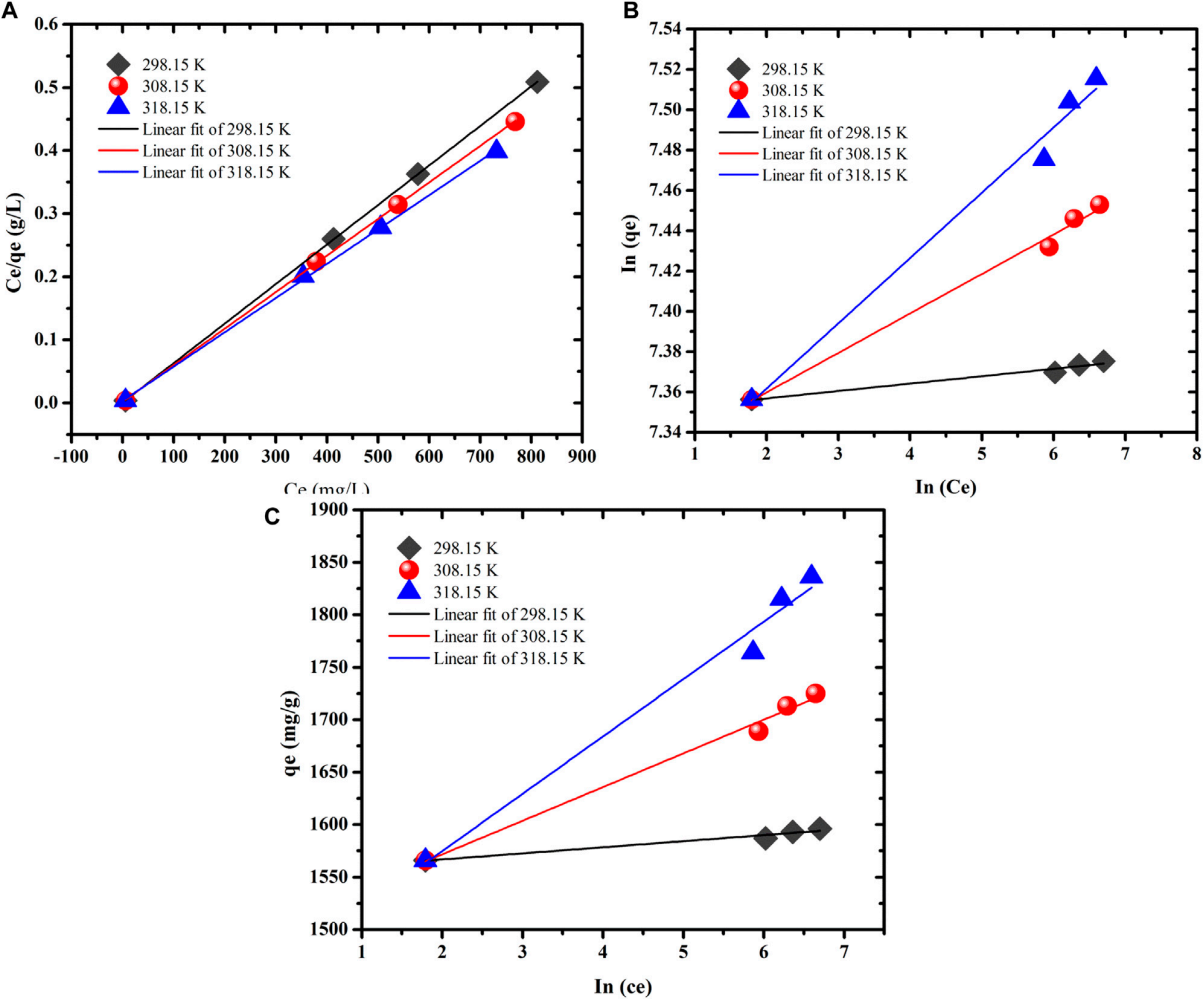
Adsorption isotherm models for MB dye onto SA/p(AAc)/*o*-MWCNTs HNC **(A)** Langmuir, **(B)** Freundlich, and **(C)** Temkin model.

To understand the relationship between the initial MB concentration and SA/p(AAc)/*o*-MWCNTs HNC the experimental adsorption data were fitted directly to three isotherm models namely Langmuir, Freundlich and Temkin models Figures 7A–C, respectively. The related isotherm parameters were calculated and tabulated in Table 2. The best-fitting adsorption isotherm model was selected based on the correlation coefficient value. The Langmuir isotherm model was the best fit for describing the adsorption process, which assumes that the adsorption process was monolayer coverage and the adsorption site on the SA/p(AAc)/*o*-MWCNTs HNC surface was homogeneous. The maximum adsorption capacities determined by Langmuir isotherm were found to be 1,596.0, 1,673.9, and 1,838.9 mg/g at 298.15, 308.15, and 318.15 K, respectively. The applicability of the Langmuir isotherm model was supported by the values of R_L_, which were less than 1 for the sorption of MB dye. Moreover, the RMSE values for Langmuir isotherm were found to be least among other studied other isotherm models, confirming that the Langmuir isotherm model is an excellent one. A brief list of the reported adsorbents, used for the removal of MB from aqueous solution as well as the obtained maximum adsorption capacity, including the prepared adsorbents in this study, is presented in Table 3 (Mittal et al., 2015; Sun et al., 2015; Mittal and Ray, 2016; Dai et al., 2017; Mahdavinia et al., 2017; Makhado et al., 2018a; Makhado et al., 2018b; Hassan et al., 2019; Ribeiro et al., 2019; Makhado et al., 2020). These results suggest that the SA/p(AAc)/*o*-MWCNTs HNC may be applied for the adsorption of MB from the aqueous phase. Moreover, SA/p(AAc)/*o*-MWCNTs HNC exhibited improved adsorption capacity as compared to unmodified hydrogel as shown in Figure 5D. SA/p(AAc)/*o*-MWCNTs HNC showed better maximum adsorption capacity than the one reported for SA/p(AAc) hydrogel (Makhado et al., 2020).

**TABLE 2:** Langmuir, Freundlich, and Temkin isotherm models for the MB adsorption.

Isotherm models	Isotherm constants	298.15 K	308.15 K	318.15 K
Langmuir	q_m_(mg/g)	1596.0	1673.9	1838.9
	b	1.1176	0.3079	0.1673
	R_L_	0.0017–0.0008	0.0061–0.0024	0.0112–0.0044
	R^2^	0.999	0.999	0.999
	RMSE	0.0022	0.0008	0.0013
Freundlich	1/n	0.004	0.020	0.032
	K_F_	1555.4	1511.1	1475.8
	R^2^	0.961	0.988	0.982
	RMSE	3.678	0.0003	0.0007
Temkin	b_T_ (kJ·mol^−1^)	427.4	79.83	48.41
	β (J·mol^−1^)	5.799	32.09	54.64
	R^2^	0.960	0.989	0.982
	RMSE	0.188	0.528	1.313

**TABLE 3:** Comparison of the MB adsorption capacity of adsorbent with other reported adsorbents.

Adsorbents	Q_max_ (mg/g)	References
PVP/PCMC/GO/bentonite hydrogel	172.1	Dai et al. (2017)
Magnetic PVA/laponite RD hydrogel nanocomposite	251.0	Mahdavinia et al. (2017)
Chitosan/silica/ZnO nanocomposite	293.3	Hassan et al. (2019)
Xylan/poly(acrylic acid) magnetic hydrogel nanocomposite	438.6	Sun et al. (2015)
XG-cl-pAA/o-MWCNTs hydrogel nanocomposite	521.0	Makhado et al. (2018b)
XG-cl-pAA/rGO hydrogel nanocomposite	793.6	Makhado et al. (2018a)
AGMOF@AA superabsorbent hybrid	1,097.4	Ribeiro et al. (2019)
SA-poly(AA)/ZnO HNC	1,129.0	Makhado et al. (2020)
TiO_2_NP-containing Gg-cl-PAAm composite hydrogel	1,305.5	Mittal and Ray (2016)
GK-cl-(PAA-co-AAM)/SiO_2_ hydrogel nanocomposite	1,408.7	Mittal et al. (2015)
SA-poly(AA)/ZnO HNC	1,529.6	Makhado et al. (2020)
SA/p(AAc)/*o*-MWCNTs HNC	1,596.0	Present work

### Adsorption Thermodynamics

**FIGURE 8:**
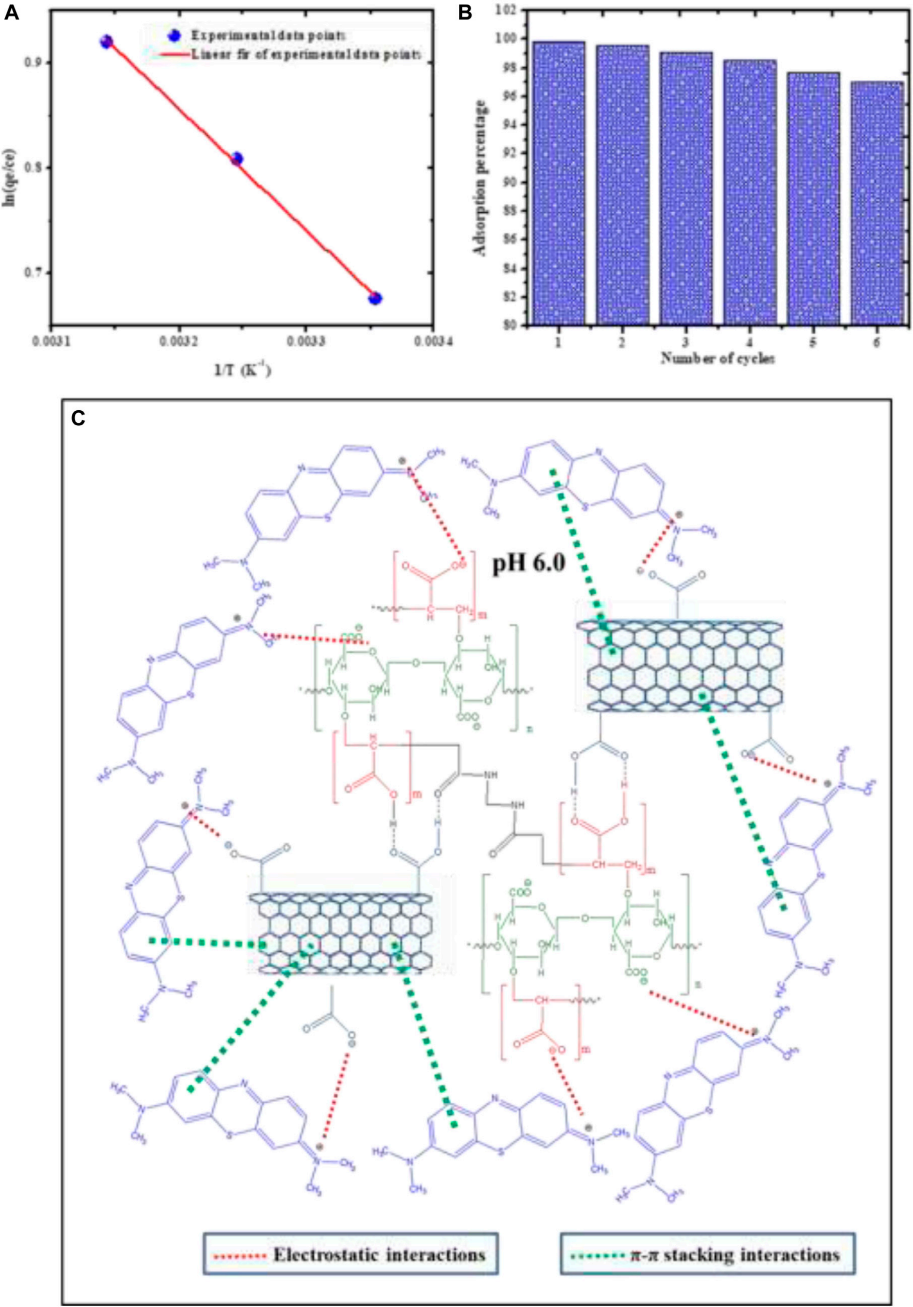
**(A)** In (q_e_/c_e_) vs. 1/T, **(B)** The regeneration of adsorbent for six-cycles adsorption/desorption of MB dye, and **(C)** Plausible mechanism for SA/p(AAc)/*o*-MWCNTs HNC and MB dye interaction.

The spontaneity of the MB dye adsorption using the prepared SA/p(AAc)/*o*-MWCNTs HNC at a temperature ranging from 298.15 to 318.15 K was evaluated to obtain thermodynamic parameters. Thermodynamics parameters such as enthalpy change (∆H°), entropy change (∆S°), and Gibbs free change (∆G°) were investigated via Vant Hoff’ equation, which is expressed as shown in Eqs 16,17:

$$\Delta {\text{G}}^{{}^{\circ}}=\Delta {\text{H}}^{{}^{\circ}}-\text{T}\Delta {\text{S}}^{{}^{\circ}},$$ (16)

$$\text{ln}\left(\frac{{\text{q}}_{e}}{{\text{C}}_{e}}\right)=\frac{\Delta {\text{H}}^{{}^{\circ}}}{\text{RT}}-\frac{\Delta {\text{S}}^{{}^{\circ}}\;}{\text{R}},$$ (17)

where R is a universal gas constant (8.314 J/mol K), T is the temperature (K) and (qe/ce) is equilibrium constant. The values of ∆H° and ∆S° were calculated from the slope and intercept of the linear plot between In (q_e_/c_e_) and 1/T shown in Figure 8A, the values of ∆G^°^ were obtained from Eq. 16 and all thermodynamic parameters are summarized in Supplementary Table S1. The positive value of ∆S° and ∆H° indicated that the adsorption process was disordering and randomness at the solid-liquid interface on the adsorbent. Improved sorption efficiency, as well as the endothermic process, was validated by the positive value of ∆H°. The negative ∆G° values showed the spontaneous nature of the sorption process (Dogan et al., 2009). Moreover, the decrease in values of ∆G° with increasing temperature suggests that the sorption is favorable at high temperatures.

### Regeneration Studies

The desorption of MB dye molecules adsorbed on the surface of SA/p(AAc)/*o*-MWCNTs HNC was conducted using HCl solution. The applicability of the hydrogel nanocomposite was investigated in six adsorption-desorption cycles and the results are shown in Figure 8B. The obtained results showed that the SA/p(AAc)/*o*-MWCNTs HNC maintained high adsorption percentage even after the sixth successive cycles under the same conditions. In conclusion, SA/p(AAc)/*o*-MWCNTs HNC can be used repeatedly for adsorption of MB dye without a significant decrease in MB dye affinity. This adsorbent holds great potential for remediation of MB wastewater.

### Adsorption Mechanisms

Figure 8C shows the plausible mechanism for the adsorption of MB dye onto SA/p(AAc)/*o*-MWCNTs HNC. Both hydrogel and filler (*o*-MWCNTs) consist of carboxyl groups on the surface. Therefore, the formation of the SA/p(AAc)/*o*-MWCNTs HNC was achieved via strong hydrogen bond between hydrogel and *o*-MWCNTs. The adsorption studies (effect of solution pH) showed that high adsorption of MB onto the SA/p(AAc)/*o*-MWCNTs HNC surface was achieved at pH range 5–9. This result suggests strong electrostatic attraction between the adsorbent and the MB dye molecules. Abundant of carboxyl groups on the surface of SA/p(AAc)/*o*-MWCNTs HNC dissociated into carboxylate anions, which influenced attraction of positively charged MB dye molecules. The π-π stacking interaction between MB onto *o*-MWCNTs may also be responsible for the high sorption capacity of SA/p(AAc)/*o*-MWCNTs HNC.

## Conclusion

In this work, the synthesis of SA/p(AAc)/*o*-MWCNTs HNC was successfully achieved via graft co-polymerization process. Different analytical techniques confirmed the presence of *o*-MWCNTs within the hydrogel network. The right degree of o-MWCNTs distribution in the polymeric hydrogel matrix was evident from TEM and SEM analysis. Moreover, the FTIR analysis showed the reduction in the absorption intensity of SA/p(AAc) hydrogel after the incorporation of *o*-MWCNTs confirming that the internal hydrogen bonding occurred among the carboxyl groups. The prepared SA/p(AAc)/*o*-MWCNTs HNC was applied as an adsorbent material for the removal of MB from aqueous solution via batch mode. The adsorption of MB onto adsorbent was influenced by several parameters including initial pH, adsorbent dose, contact time as well as equilibrium concentration. The adsorption kinetics and isotherm model revealed that the sorption process was best described by pseudo-second-order and the Langmuir models, respectively. Under optimal condition, the maximum adsorption capacity of SA/p(AAc)/*o*-MWCNTs HNC for MB dye was found to be 1,596.0 mg/g at 25°C obtained from the Langmuir isotherm model. The regeneration experiments demonstrated that the prepared adsorbent could be applied repeatedly for several cycles without significant reduction in the adsorption capacity. The adsorption studies showed that the electrostatic interaction was the main driving force for the sorption process. The SA/p(AAc)/*o*-MWCNTs HNC prepared in this study holds great potential for implementation in wastewater treatment.

## Data Availability

The raw data supporting the conclusions of this article will be made available by the authors, without undue reservation.
