# Supplementary material for: Preparation and Characterization of Sodium Alginate-Based Oxidized Multi-Walled Carbon Nanotubes Hydrogel Nanocomposite and its Adsorption Behaviour for Methylene Blue Dye
Source: Front Chem. 2021 Mar 17;9:576913. doi: 10.3389/fchem.2021.576913 (PMC8009996; doi:10.3389/fchem.2021.576913)
Supplement: Supplementary file 1 [file table1.docx]

**ELECTRONIC SUPPLEMENTARY MATERIAL**

**Manuscript ID: 576913**

**Preparation and Characterization of Sodium Alginate-based Oxidized Multi-Walled Carbon Nanotubes Hydrogel Nanocomposite and its Adsorption Behaviour for methylene blue**

*Edwin Makhado** *and Mpitloane Joseph Hato**

*Nanotechnology Research Lab, Department of Chemistry, School of Physical and Mineral*

*Sciences, University of Limpopo (Turfloop), Sovenga 0727, Polokwane, South Africa*

**
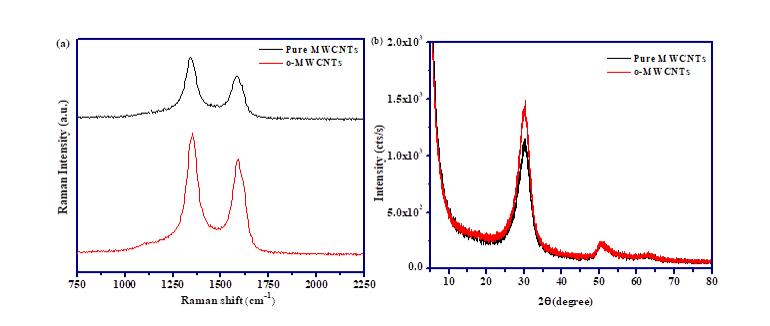
**

**FIGURE S1** **(a)** Raman spectra and **(b)** XRD patterns of pure MWCNTs and *o*-MWCNTs.

**TABLE S1** Thermodynamic parameter for the sorption of MB onto SA/p(AAc)/o-MWCNTs hydrogel nanocomposite.

| **Adsorbent** | **Temperature (K)** | **∆G° (kJ mol^-1^)** | **∆H° (kJ mol^-1^)** | **∆S° (kJ mol^-1^ K^-1^)** | |
| --- | --- | --- | --- | --- | --- |
| SA/p(AAc)/o-MWCNTs HNC | 298.15 | -1.711 | 9.619 | 0.038 |  |
|  | 308.15 | -2.091 |  |  |  |
|  | 318.15 | -2.471 |  |  |  |


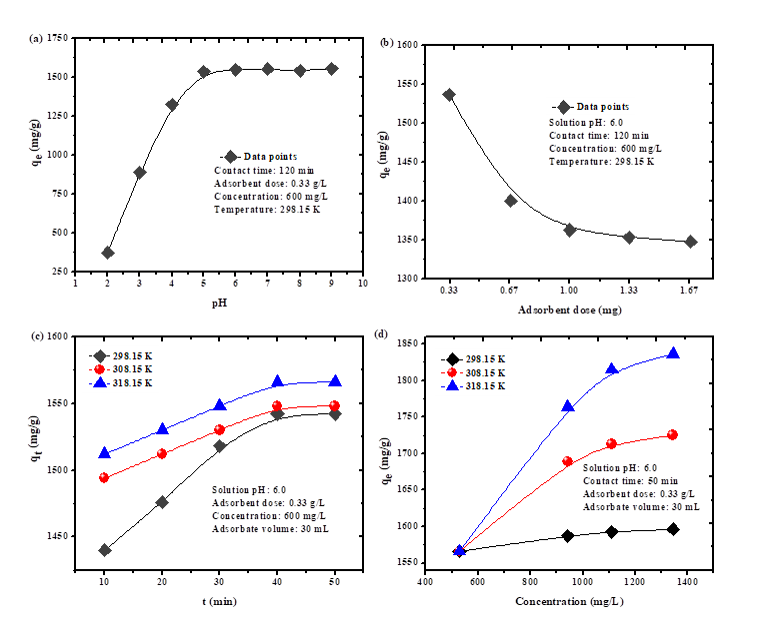


**FIGURE S2** Effect of **(c)** contact time, and **(d)** equilibrium concentration on the adsorption capacity of MB dye at three different temperatures
