# Supplementary material for: Preparation and Characterization of Sodium Alginate-Based Oxidized Multi-Walled Carbon Nanotubes Hydrogel Nanocomposite and its Adsorption Behaviour for Methylene Blue Dye
Source: Front Chem. 2021 Mar 17;9:576913. doi: 10.3389/fchem.2021.576913 (PMC8009996; doi:10.3389/fchem.2021.576913)
Supplement: Supplementary file 2 [file table2.docx]

**REVIEWERS COMMENTS**

**Manuscript ID: 576913**

**Preparation and Characterization of Sodium Alginate-Based Oxidized Multi-Walled Carbon Nanotubes Hydrogel Nanocomposite and its Adsorption Behaviour for Methylene Blue**

***Edwin Makhado** *and Mpitloane Joseph Hato****

*Nanotechnology Research Lab, Department of Chemistry, School of Physical and Mineral Sciences, University of Limpopo (Turfloop), Sovenga 0727, Polokwane, South Africa*

Thank you for granting us the opportunity to submit a revised manuscript with the title “**Preparation and Characterization of Sodium Alginate-Based Oxidized Multi-Walled Carbon Nanotubes Hydrogel Nanocomposite and its Adsorption Behaviour for Methylene Blue ”** for publication as a Special Issue in ***Frontiers in Chemistry: Rising Star Africa***. We appreciate the time and effort that the reviewers dedicated to providing feedback on our manuscript and are grateful for the insightful comments and valuable improvements to our paper. We have incorporated all the suggestions to the best of our abilities. The changes are highlighted in RED, for a point-by-point response to reviewers’ comments and concerns.

Response to Reviewers' comments

**Reviewer 2**

1.The author should prove the novelty of the work this is not accepted

Response: The study showed that the incorporation of a functionalized MWCNTs into the sodium alginate/poly(acrylic acid) hydrogel has a greater influence on various properties of the hydrogel such as an adsorptive performance in comparison with bare SA/p(AAc) hydrogel and other reported adsorbents in the literature. Moreover, the incorporation of oxidized-MWCNTs in the polymeric hydrogel improved properties such as thermal stability as well as swelling capacity. This study lead to better understanding of fundamentals in the field of hydrogel nanocomposites for adsorption of contaminants. The findings from this study will give other researchers a better understanding of the behavior of hydrogel nanocomposites for adsorption of other dye contaminated wastewater.

2. Minor errors like

Response: We have thoroughly revised the entire manuscript and corrected all errors to the best of our abilities.

3. Use of abbreviation in the entire paper without defining them–throughout

Response: As per reviewer’s suggestion, an abbreviations are defined when they appear at the first time in the revised manuscript.
4. No use of statistics

Response: The experimental data points of figures with error bars are shown in the revised manuscript.
5. No reproducibility

Response: The desorption and regeneration studies were conducted and the results are shown in Figure 8b.
6. Authors should use another dye and should prove the capacity of the material- Mixture is also recommended

Response: At this stage we could not conduct a series of experiments due to COVID-19 strict regulation on campus. We will definitely consider reviewer’s valuable suggestion and conduct a series of experiments with other dyes or a mixture in further studies as proposed by the reviewer.
7. Most of the fig can be moved in supplementary

Response: Thanks, several figures are moved to supplementary file.
8. A clear cut comparison is expected between earlier and current developed material

Response: Thanks for a valuable suggestion, we have provided the justification by providing the following text provided below in the main script along with the comparative Table 3.

A brief list of the reported adsorbents, used for the removal of MB from aqueous solution as well as the obtained maximum adsorption capacity, including the prepared adsorbents in this study is presented in **Table 3** (Dai et al., 2018; Mahdavinia et al., 2017; Hassan et al., 2019; Sun et al., 2015; Makhado et al., 2018a; Makhado et al., 2018b; Ribeiro et al., 2019; Mittal and Ray, 2016; Mittal et al., 2015; Makhado et al., 2020). These results suggest that the SA/p(AAc)/*o*-MWCNTs HNC may be applied for the adsorption of MB from the aqueous phase. Moreover, SA/p(AAc)/*o*-MWCNTs HNC exhibited improved adsorption capacity as compared to unmodified hydrogel as shown in **Figure** **5(d)**. SA/p(AAc)/*o*-MWCNTs HNC showed better maximum adsorption capacity than the one reported for SA/p(AAc) hydrogel (Makhado et al., 2020).

**Reviewer 3**

Comments to 576913：
This study prepared sodium alginate/oxidized multi-walled carbon
nanotubes-derived hydrogel nanocomposite for adsorbing methylene blue and characterized employing various characterization techniques. After careful review, this manuscript needs to be largely improved before further consideration.

1.In the synthesis of SA/p(AAc)/o-MWCNTs HNC, the font in the first sentence is different from that in the following part.

Response: We greatly appreciate the valuable suggestion of the reviewer for improving the quality of the manuscript. The font in the first sentence is corrected.

2.The prepared adsorbent is in powder form. Is it easily recovered?

Response: Yes, the prepared adsorbent was in a powder form and the adsorbent is recovered by filtration.
3.In line 377, it should be the o-MWNT that causes the decrease in G'.

Response: We greatly appreciate the reviewer’s valuable suggestions offered. As suggested by the reviewer 3, we have corrected line 377 and now reads “the o-MWCNTs caused the decrease in G'”

4.According to the position of point in Fig.4c,the maximum swelling percentage of the adsorbent is at pH 8,while it is written in the manuscript that the swelling percentage reaches its maximum at pH 9.

Response: Thanks, correction made and it is written that the maximum swelling percentage was reached at pH 8.

5.Why does this adsorbent generate negative charge density which can interact statically with water molecules in the alkaline environment?

Response: At pH above 6 most of the carboxylic groups are ionized and electrostatic interaction between the adsorbent surface (COO^­­-^ ) and the cationic MB dye and/or water molecules is responsible for high adsorption efficacy.

6.When explaining the effect of pH on adsorption, it is best to compare to Zeta potential.

Response: Thanks for a valuable suggestion, pH_pzc_ was studied and compared with the effect of initial pH on adsorption experiments.

7.It can be seen from Fig.5a that the adsorption capacity is almost the same at pH 6 or 7. Why did the authors choose pH 6 as the experimental condition after that?

Response: Based on experimental optimization conditions, high adsorption capacity was noticed at pH 6. Hence this pH was used as an optimum initial pH during adsorption processes.

8.Why does the adsorption capacity decrease when the pH is higher than 6?

Response: The adsorption capacity increased from low pH to pH 6 and remained almost constant when the pH was higher than 6.

9.These images that systematically examined the effects of key parameters such as initial pH, adsorption dose, contact time and concentration on the adsorption capacity should be compared with unmodified adsorbent.

Response: Thanks, Figure 5 shows that the effect of initial pH, adsorption dose, contact time and concentration on the adsorption capacity of SA/p(AAc)/*o*-MWCNTs HNC which was compared with unmodified SA/p(AAc) hydrogel.

10.The experimental data points of all figures should show error bars in the figures using three experimental repetitions. And the resolution of all figures should be improved.

Response: Thanks for the suggestion, the experimental data points of figures show error bars and the resolution of figures have been improved.

11.Please confirm that ordinate in Fig.8a is displayed correctly.

Response: Yes, the ordinate in Figure 8a is presented correctly.

12.How to treat the adsorbent after the experiment?

Response: Authors give their thanks to the learned reviewer for a comment, as given as in the form of valuable suggestion. The adsorbent will be treated as follow after the experiments:

**Desorption and Regeneration Studies**

“Reusability of samples was studied through repeated adsorption-desorption experiments. The MB-loaded adsorbents were recovered and recycled by rinsing with 0.1 M HCl solution and agitated at 170 rpm for 3 hrs, then neutralized with 0.1 M NaOH solution followed by DI water. The absorbent were dried at 50 $^{\circ}$C, pulverized, and reused in the next cycle of the adsorption. The dye adsorption/desorption cycles were repeated six times and calculated as given in Equation (16).

Desorption percentage (%) = $\frac{Concentration desorbed (mg/l)}{Concentration adsorbed (mg/l)}$ x 100	(16)”

13.English writing should be highly improved.

Response: The authors have thoroughly revised the entire manuscript and corrected the language, grammar and typological errors to the best of their abilities.
